# Prevalence of Antiretroviral Drug Resistance Mutations and HIV-1 Subtypes among Newly-diagnosed Drug-naïve Persons Visiting a Voluntary Testing and Counselling Centre in Northeastern South Africa

**DOI:** 10.3329/jhpn.v29i4.8444

**Published:** 2011-08

**Authors:** Julius M. Nwobegahay, Pascal O. Bessong, Tracy M. Masebe, Lufuno G. Mavhandu, Benson C. Iweriebor, Gloria Selabe

**Affiliations:** ^1^AIDS Virus Research Laboratory, Department of Microbiology, University of Venda, PMB X5050, Thohoyandou 0950, South Africa; ^2^HIV/AIDS and Hepatitis Unit, Department of Medical Virology, University of Limpopo, MEDUNSA Campus, Pretoria, South Africa

**Keywords:** Antiretrovirals, Drug resistance, Drug-naïve persons, HIV-1, South Africa

## Abstract

Data on antiretroviral drug resistance among drug-naïve persons are important in developing sentinel surveillance policies. This study was conducted to determine the prevalence of antiretroviral drug resistance mutations among drug-naïve HIV-infected individuals attending a voluntary testing and counselling centre at the Mankweng Hospital in northeastern South Africa. In total, 79 drug-naïve HIV-positive individuals were sequentially recruited during February 2008–December 2008. Drug resistance mutations were determined using the calibrated population resistance tool available on the Stanford HIV drug resistance database. Viral DNA was obtained from 57 (72%) of the 79 individuals. Reliable nucleotide sequences were obtained for 54 reverse transcriptase (RT) and 54 protease (PR) gene regions from 54 individuals. Overall, five sequences (9.3%) harboured drug resistance mutations (95% confidence interval −1.53 to 16.99). Four (7.4%) of these were nucleoside RT inhibitor mutations (D67G, D67E, T69D, and T215Y), and one (1.9%) was a PR inhibitor mutation (M46I). No major non-nucleoside RT resistance mutation was detected. Several minor resistance mutations and polymorphisms common in subtype C viruses were observed in the PR and RT genes. Phylogenetic analysis of the partial *pol* sequences showed that 52 (96%) of the 54 isolates were HIV-1 subtype C. One isolate (08MB08ZA) was HIV-1 subtype B while another (08MB26ZA) was related to HIV-1 subtype J. HIV-1 subtype recombination analysis with REGA assigned the *pol* sequence to HIV subtype J (11_cpx) with a bootstrap value of 75%. The prevalence of drug resistance mutations observed in the population studied was relatively higher than previously reported from other parts of South Africa. In addition, this is apparently the first report of an HIV-1 subtype J-like virus from northeastern South Africa.

## INTRODUCTION

The health and quality of life of patients infected with HIV/AIDS have dramatically improved since the introduction of highly-active antiretroviral therapy (HAART) in 1996 (1,2). However, one of the major drawbacks of HAART is the development of drug resistance which usually accompanies the use of antiretrovirals (ARVs) (2,3). Drug-naïve individuals with ARV drug resistance have a relatively higher risk of virologic failure as they start antiretroviral treatment (ART) with a lower genetic barrier to resistance (4). In addition, harbouring resistance viruses before the initiation of treatment complicates alternative treatment algorithms.

In the developed world, HAART has been used with remarkable success, and treatment is accompanied with regular virologic monitoring, including measurements of viral load and testing of drug resistance to guide management of patients (5,6). On the other hand, the use of HAART in resource-poor settings still poses a lot of problems. Virological monitoring is less frequently done due to involvement of cost and dearth of qualified personnel.

South Africa has one of the highest HIV/AIDS prevalence in the world, and the South African National HIV and Syphilis Survey in 2007 showed that the estimated prevalence was 28% (7), with the Limpopo province (northeastern South Africa) having a prevalence of 20.7%. Before 2004, ARVs were available only through private health establishments in South Africa, and due to the scarcity and prohibitive high cost, the majority of patients could not afford treatment. In early 2004, a national ARV delivery programme was initiated to provide drugs to those who had a CD4 count of <200 cells/μL or with a clinically-defined AIDS condition. Before 2010, the South African ARV drug regimen guideline recommended the use of stavudine, lamivudine, and efavirenz (with nevirapine replacing efavirenz for women of childbearing age). Currently, the revised regimen comprises tenofovir, lamivudine, and efavirenz/nevirapine. Stavudine is still recommended where side-effects are tolerated (8).

The determination of a regimen based on the genetic resistance profiles of patients’ viruses before the initiation of therapy generally correlates with a better outcome compared to the absence of such resistance data (3,9). Information on the presence of resistant viruses is important in tailoring combination regimens or to decide whether to prescribe testing of resistance before the initiation of therapy. It was estimated that 500,000 adults and children were receiving ARVs in South Africa by mid-2008, with about 34,000 of them in Limpopo province (northern South Africa) (10). In South Africa, some studies have indicated the prevalence of genetic drug resistance among the naïve population of less than 5% (9,11). However, despite the low prevalence reported, it is important to monitor the prevalence of drug resistance in drug-naïve populations, particularly in regions, such as northeast of South Africa where such data are scarce. The only data on resistance available for northern South Africa were obtained from samples collected in 2001 from Waterberg district (10,12) before the availability of ARVs through the public health sector in 2004. Findings of these studies showed the absence of resistance mutations in the samples studied. In addition, since 2004, access to ARVs has escalated in northern South Africa (from an estimated 300 in 2001 to 34,000 in 2008) (11), and it is important to address development of drug resistance through periodic studies. The aim of this study was, therefore, to provide baseline data on ARV drug resistance mutations in Mankweng (Capricorn district), one of the areas where ART first began in northeastern South Africa in 2004 and where no resistance data are available. Information generated could be useful in developing sentinel surveillance policies for the region.

## MATERIALS AND METHODS

### Study population, sample collection, and plasma preparation

Individuals without prior exposure to ARVs and who tested positive for HIV antibodies at the HIV Voluntary Testing and Counselling Centre at the Mankweng Hospital, South Africa, were recruited sequentially during February 2008–December 2008. Viral load and CD cell counts are not routinely measured at the clinic, and data on these were not available. Participants were apparently healthy individuals at the time of HIV testing. The Mankweng Hospital is a component of the Polokwane-Mankweng Hospital Complex, a regional referral hospital for Limpopo province in northern South Africa. The Voluntary Testing and Counselling Centre at the Mankweng Hospital caters to 19 health centres in the rural precincts of Mankweng.

Five mL of venous blood was collected into an EDTA vacutainer tube from each consenting subject. A questionnaire was used for collecting demographic data, such as age, sex, place of residence, probable date and place of infection, and marital status. Most (87.3%) samples were processed for total RNA extraction within 48 hours while stored at 4 °C. However, 10 samples, due to transportation logistics, were stored at 4 °C for four days before RNA isolation. Whole blood was spun at 3,000 rpm for three minutes. Plasma was aspirated aseptically and stored at −80 ˚C for subsequent RNA extraction. Data on viral load and CD4 count were not available.

### Isolation of viral RNA, RT-PCR, and nested PCR

Viral RNA was isolated using the viral mini RNA kit (Qiagen, Hilden, Germany) and stored at −80 °C until used. Two protocols were used at different times for amplifying the HIV polymerase gene. In one protocol, a one-tube RT-polymerase chain reaction (RT-PCR) was performed with AMV RT (Roche, USA) and Taq DNA polymerase (Invitrogen), followed by a nested PCR as previously described (10). This generated approximately a 1.4-kb product comprising the entire PR and at least 900 bp of the RT gene. In the other protocol, cDNA was synthesized using the Thermoscript RT-PCR system (Invitrogen, Hilden, Germany). Thereafter, a 1.7-kb fragment of the polymerase gene comprising the entire PR and at least 1200 bp of the RT gene was amplified using the Expand Long Template System (Roche) as previously described (11). The PCR products were verified for expected size by electrophoresis of 1% agarose gel stained with ethidium bromide. PCR amplicons were purified with QIAquick PCR purification kit (Qiagen, Hilden, Germany), and direct population-based sequencing was performed on both strands with the BigDye Terminator v3.0 kit on ABI Prism 377 (Applied Biosystems) using Taq DNA polymerase. Generated nucleotide sequences were edited manually using the SeqMan II software (version 7) (DNASTAR, Lasergene, USA).

### Viral genetic subtyping

Subtyping of HIV was done by phylogenetic analysis. Polymerase sequences (880 bp) comprising the complete PR and partial RT genes of test viruses were aligned using ClustalX with representative subtype reference sequences (group M subtypes A-D, F-H, J, and K, excluding recombinant sequences) obtained from GenBank (13). Previously-described sequences from Limpopo province were included in analysis. Neighbour-joining phylogenetic trees were generated with the PHYLIP programme, making the use of the maximum likelihood model to take care of differences in the transition and transversion rates. Trees were rooted with an HIV-1 group O strain (L20571). The reliability of the trees was assessed by a bootstrapping of 1,000 replicates. REGA HIV-1 and 2 subtyping tool (version 2.0) was used for analyzing the sequences for recombination (14,15). The tool employs phylogenetic analysis and bootscanning methods to assign genetic subtypes. Predicted amino acids of the PR and RT genes were aligned using the BioEdit program (16). Consensus amino acid sequences of the PR and RT regions were created and then compared with the global subtype C and the global subtype B consensus sequences obtained from GenBank (2000). The mean genetic distances among the PR and RT sequences were determined by the Kimura 2parameter model.

### Determination of genetic drug resistance

Drug resistance mutations were examined according to the calibrated population resistance (CPR) tool (version 5.0) beta contained in the Stanford HIV Drug Resistance database (http://cpr.stanford.edu/cpr/servlet/CPR). The CPR tool analyzes submitted sequences for resistance mutations based on the surveillance transmitted drug resistance mutations list of the World Health Organization (17). The tool also detects unusual, bad and hyper mutated sequences and performs assignment of subtype.

### Ethical considerations

The Health, Safety, and Research Ethics Committee of the University of Venda, South Africa, approved the study protocol. The Limpopo Provincial Department of Health and the Mankweng Hospital permitted to conduct the study. Signed informed consent was obtained from each study participant.

## RESULTS

### Demographics of study subjects

In total, 79 HIV-positive individuals were sequentially recruited for the study. Their mean age was 38.5 (range 18-59) years. The proportion of women and men was 73.4% and 25% respectively. About 80% of the study subjects were single, and 22.8% were married. The most important risk factor for HIV transmission based on responses to the questionnaire was sexual intercourse (97.5%). However, the questionnaire was not designed to determine whether it was heterosexual or homosexual. In 89.9% of the participants, the most probable place of HIV infection was South Africa. About 47% of the subjects were infected in 2008 while the remaining subjects estimated that they were infected during 2000-2007. The highest level of education for 79.7% of the participants was grade 6 and above but no university graduates were recorded within the participants. Details of the demographic and socioeconomic characteristics of the study population are shown in the Table.

### Viral DNA amplification drug resistance mutations

Viral DNA was obtained for 57 (72%) of the 79 individuals. Reliable nucleotide sequences were obtained for 54 RT genes and 54 PR genes, on which genetic subtyping and drug resistance mutation analyses were done. Using the CRP analysis for surveillance drug resistance mutations, five (9.3%) of the 54 sequences had mutations associated with drug resistance (95% confidence interval −1.53 to 16.99). These included four NRTI mutations (D67E, D67G, T69D, and T215Y) harboured by sequences 08MB38ZA, 08MB26ZA, 08MB27ZA, and 08MB29ZA respectively, and one PI resistance mutation (M46I) observed in sequence 08MB62ZA. No NNRTI mutation was detected, and no sequence harboured more than one resistance mutation.

### HIV genetic subtypes

**Fig. 1.**
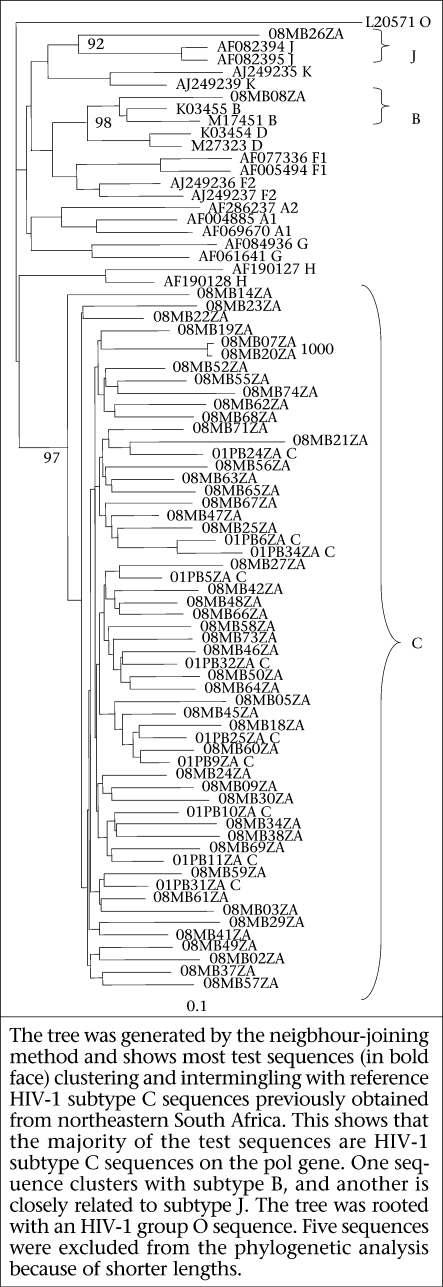
Phylogenetic analysis of partial HIV pol sequences from drug-naïve individuals from northeastern South Africa

**Fig. 2.**
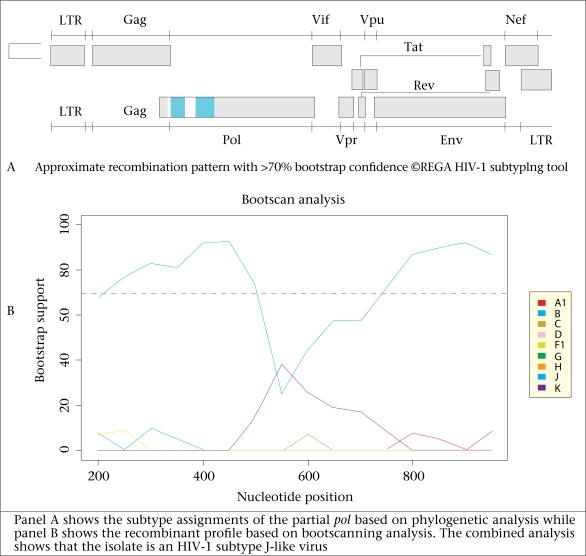
Subtype assignment and HIV-1 subtype recombination analysis of 08MB26ZA

Phylogenetic analysis of the partial *pol* sequences showed that 52 (96.3%) of the 54 isolates were HIV-1 subtype C. One isolate (08MB08ZA) was HIV-1 subtype B while isolate 08MB26ZA was observed to be related to HIV-1 subtype J reference sequences (Fig. 1). Bootscanning analysis of 08MB26ZA with the REGA HIV-1 subtyping tool showed that its *pol* gene has a mosaic structure and was related to CRF_11 (Fig. 2). The STAR genotyping feature with the CRP analysis also assigned 08MB26ZA to CRF_11. Futhermore, this sample harboured the only D67G NRTI mutation observed in the study. Pure subtypes were assigned to all the other sequences after bootscanning analysis. The mean genetic distance for the PR sequences ranged from 0.0239 to 0.2519 and 0.0273 to 0.1527 for the RT sequences. Considering the first 300 amino acids in the RT gene, sequence alignment showed that the consensus of the test viruses was identical to the global subtype C consensus. It differed from the global subtype B consensus at 18 positions (V35T, E36A, T39E, S48T, K122E, D123G, K173A, D177E, T200A, Q207E, R211K, V245Q, A272P, K277R, T286A, E291D, V292I, and I293V). Amino acid alignment of the PR gene showed that the test consensus was identical to the global subtype C consensus, except at position T13I. It differed from the global subtype B consensus at eight positions (T12S, I15V, L19I, M36I, R41K, H69K, L89M, and I93L).

**Table.** Demographic and socioeconomic characteristics of study population

Characteristics	Patients (n=79)	
	No.	%
Sex		
Male	20	25.3
Female	58	73.4
Data not available	01	1.3
Marital status		
Single	56	70.9
Married	18	22.8
Widowed	01	1.3
Divorced	02	2.5
Data not available	02	2.5
Probable place of infection		
South Africa	71	89.9
Other countries	00	0
Data not available	8	10.1
Risk factor for transmission		
Sex	77	97.5
Data not available	02	2.5
Probable year of infection		
Before 2000	00	0
2000-2007	40	50.6
2008	36	45.6
Data not available	03	3.8
Household income (US$)		
<150	59	74.7
150-500	18	22.8
>500	00	0
Data not available	02	2.5
Number of dependants		
1-5	58	73.4
≥6	04	5.1
Data not available	17	21.5
Highest level of education		
Not educated	02	2.5
≤Grade 5	04	5.1
Grade 6 and above	63	79.7
University	06	7.6
Data not available	04	5.1

### Sequence accession numbers

The PR and RT gene sequences reported here have been submitted to GenBank with the following accession numbers: PR: GU188807-GU188752; RT: GU188753-GU188806.

## DISCUSSION

The development of drug resistance is an important attribute of HIV. Infection with drug-resistant viruses complicates the clinical management of patients. Due to the emergence of resistant viruses, surveillance of drug-resistant variants among the population is necessary as this may inform the establishment of sentinel surveillance or genotypic resistance testing before the initiation of therapy (3,8,17,18).

Antiretroviral treatment was started at the Mankweng community in 2004, four years before the collection of samples, within which time there is a possibility of emergence of resistance and transmission. Thus, the aim of the present study was to determine the prevalence of drug resistance-associated mutations in drug-naïve individuals in Mankweng, South Africa. The study participants were individuals who ‘walked’ into the HIV Voluntary Testing and Counselling Centre for the first time. Blood samples were collected sequentially from individuals who tested positive. Resistance-associated mutations were detected in five of the 54 analyzed subjects, giving a prevalence of patients with resistant viruses of 9.3%. Only NRTI and PI resistance mutations were detected. According to the WHO resistance classification, a prevalence of 9.3% is moderately high (5-15%). Furthermore, the observed prevalence was also relatively higher compared to what has been previously reported from other parts of South Africa. For example, a surveillance study of drug resistance mutations in naïve persons in Gauteng province in 2002 and 2004 reported a 4.2% prevalence (9). In another study in Cape Town (Western Cape province), the prevalence of major drug resistance mutations in naïve patients was 2.5% (11). Similarly, a prevalence of 3.6% has been reported from the Free state (19). Low levels (<5%) or the absence of drug resistance mutations have been reported in other southern African countries, such as Zambia (20) and Malawi (21). The prevalence observed in the present study is similar to what has been reported in some high-income countries with more than a decade of ART-use. Some of these countries include Britain, where a 14.2% resistance level (22) and the United States where up to 25% have been documented (23). Unlike in high-income countries, the low prevalence of drug resistance mutations was expected in low-income countries in the early phase of treatment roll-out because most patients were starting therapy on highly-potent regimens. In high-income countries, ART-use began with resistance-associated monotherapy and one-class dual therapy (24).

In the present study, all the observed resistance mutations were detected in different individuals, with 2008 being the probable year of infection. Mutations in D67E, D67G, and T69D were detected in three women while T215Y and M46I were detected in two men. Several polymorphisms, such as K20R, M36I, and I93L, common in HIV-1 subtype C viruses and associated with drug resistance site, were also observed as has been previously reported (11). According to the WHO drug resistance algorithm, substitution D67E has the potential to cause low-level resistance to zidovudine, stavudine, and didanosine while D67G causes low-level resistance to zidovudine, and a potential for low-level resistance to abacavir, stavudine, and didanosine. In the same vein, T69D causes low-level resistance to didanosine, with a potential for low-level resistance to stavudine. Substitution T215Y causes intermediate resistance to zidovudine and stavudine and low-level resistance to abacavir, didanosine, and tenofovir. The only protease inhibitor (PI) mutation observed —M46I—causes intermediate resistance to nelfinavir, with a potential for low-level resistance to ritonavir-boosted doses of atazanavir, fosamprenavir, indinavir, and lopinavir.

At the time of the present study, a combination regimen of stavudine, lamivudine, and efavirenz was the recommended first-line regimen, with nevirapine replacing efavirenz for women of childbearing age. A PI-containing regimen was used for children aged less than three years and in second-line regimen for adults (8).

The study participants claimed not to have been treated with ARVs before the initiation of the study. Most study patients would benefit from the current first-line treatment regimen based on their viral genetic profiles. However, it is difficult to predict the spread of mutations observed and the implications on treatment outcomes at the population level. The resistance mutations described herein were observed after four years of the availability of treatment in the public health sector in the study area. It should also be noted that, although access to treatment in the public sector began in 2004, ARVs were available through the private sector before 2004, albeit at a higher cost and limited access. Accepting that the study participants had no prior exposure to ARVs, the mutations may have been acquired from transmitted viruses. Although the participants were apparently healthy, the absence of viral load and measurements of CD4 count do not allow a good estimation of their immunologic and virologic state. Mutations may revert over time in the absence of drug pressure, leading to a possible underestimation of transmitted resistance viruses. DNA amplification could not be obtained from about 25% of the samples probably because of very low viral loads, since most patients were asymptomatic. Subsequent surveillance studies are needed to confirm the findings of this report and to generate data relevant to sentinel surveillance policy and treatment-initiation guidelines.

The identification of subtype C viruses as the predominant subtype is in agreement with findings of previous genotyping studies in northern South Africa (10,12). However, the presence of subtype B and CRF_11-related viruses has not been previously reported. Since only the partial pol gene of the CRF_11-like sample was analyzed, a full-length genomic analysis of the virus would be required to reveal its complete mosaic nature.

## ACKNOWLEDGEMENTS

The study was supported by a research grant awarded to Pascal O. Bessong by the National Department of Health and the National Research Foundation of South Africa. Permission to publish the results was obtained from the National Department of Health. The authors are grateful to Dr. C. Clark, Dr. S. Maweya, and Ms Khanyisa Sono of the Mankweng Hospital, South Africa, for recruiting the patients. The cooperation of the study participants is appreciated.
